# Experimental Researches Applied to Physiology and Pathology

**Published:** 1854-01

**Authors:** 


*Experimental Researches applied to Physiology and Pathology.* By E. Brown-Sequard, M.D., P. etc., New York. H. Bailliere, No. 290 Broadway.

We have received this little work from the publisher. The articles which it contains have already appeared in the Medical Examiner and are now collected and presented to the profession in a permanent form. The experiments of this distinguished physiologist have been regarded with much interest by the profession in this country.

The article on the treatment of epilipsy we publish entire. Our readers will remember that in our last volume we published a paper from Dr. Marshall Hall on this disease. It will be seen that our Author fully sustains Dr. Hall's views:

I have made numerous experiments with regard to the treatment of this dreadful affection, and I intend to publish them, in extenso, when some points that are still obscure have become clear to my mind. Here I will merely relate some of the most important results of my researches. As I have had the opportunity during the last three or four years of observing every day a great many animals (more than a hundred) which had a convulsive affection resembling epilepsy very much, I have been able to discover some very interesting facts, among which are the following:

1st. For each epileptic animal, the number of fits, in a given time, is generally in a direct proportion with the quantity of food taken.

2d. There is an inverse proportion between the amount of exercise and the number of fits.

3d. Cauterisation of the mucous membrane of the larynx is able either to cure or to relieve these epileptic animals.

The convulsive affection existing in almost all these animals was the consequence of a transversal section of a lateral half of the spinal cord, in the dorsal or in the lumber region.

I have already published the results of my experiments on epilepsy, in my lectures before large classes of Physicians and Medical students, both in France in 1851 and in this country in 1852.

These results are in perfect accordance with the views of Dr. Marshall Hall in relation to epilepsy. As the views of this eminent biologist are generally known, I need not expose them, and I will merely remind my readers of the three following points:

1st. The first muscles that contract spasmodically in almost all, if not all, the cases of epileptic fits, are those of the larynx and the neck; 2nd, spasms of the glottis taking place then, produces suffocation, in consequence of which convulsions are produced in the trunk and the limbs; 3d, tracheotomy may prevent these convulsions by preventing suffocation, and it is known that in some cases tracheotomy has cured epilepsy.

It has been objected to Marshall Hall that in cases of poisoning by strychnine, convulsions take place even when a tracheal tube renders respiration perfectly free. This objection has no value, because the state of the spinal cord in epileptics is not the same as in men or animals poisoned by strychnine. Certainly the excitability of the spinal cord is greater in epileptics than in healthy persons, but the degree of excitability of that nervous centre is much greater in persons poisoned by strychnine than in epileptics; and, therefore, it is easy to understand that certain excitations are [illegible]le to produce general convulsions in one case and not in the [illegible]her.

[illegible] we give a very slight dose of strychnine to an animal, so as [illegible] poison it, but merely to increase slightly the excitability of the spinal cord, there are no convulsions when we touch or pinch or burn the skin, but if we prevent breathing for a few seconds only, general convulsions take place, exactly as in epileptic men or animals.

It has been said also, in opposition to Marshall Hall, that a spasm of the glottis of the severest kind occurs in cases of hooping cough, of spasmodic croup and even of apoplexy, without the occurrence of any other convulsions. The answer to this objection is, that in epilepsy the spinal cord is more excitable than in these other diseases, so that the same kind of excitation does not produce the same effect.

A great many facts, that I will publish elsewhere, prove that black blood, very probable by its carbonic acid, is an excitant of the spinal cord and of the medulla oblongata. When, as is the case in asphyxia, the blood is not oxygenated and deprived of the carbonic acid constantly produced in it, or received by it from different tissues, then the excitation made on these nervous centres becomes so powerful that convulsions are produced. This is found in men and animals, even in perfect health. If the asphyxia is incomplete, convulsions are not produced, unless the excitability of the spinal cord is greater than usual, and this is the case in epileptics.

In November, 1851, at the *Ecole Pratique*, of Paris, I published for the first time, before a class of about forty young Physicians and Medical students, the results of my experiments as regards the cauterization of the larynx in epilepsy. About eight months after, Dr. Eben Watson published a paper* in which he says: "The treatment I would now propose instead of tracheotomy is simply the application of a solution of nitrate of silver, varying in strength with the requirements of the case, to the glottis of the patient, with the view of diminishing the nervous excitability of the part in question. A similar treatment has been found by me remarkably successful in alleviating and removing, in a short time, the susceptibility of the patient to laryngysmus, in cases of hooping cough, and of spasmodic croup (*laryngysmus stridulus*,) nor can I see any reason why a similar result should not ensue in chronic cases of epilepsy."

The reasons given by Dr. E. Watson are partly the same by which I had been led long before him to perform the operation he suggests. But I had also some other reasons. It is perfectly known, in the actual state of Medical Science, that the greatest changes may be produced in the nervous centres, as well as in the nerves, by a very strong excitation of the termination of

* Remarks on Dr. M Hall's theory of the relation of Laryngysmus to Epilepsy. In London Journal of Medicine, July, 1852, pp. 641–43.

the nervous fibres in the skin or the mucous membranes. On this principle are founded many modes of treatment of some diseases of the spinal cord and of neuralgia. The application of caustics, blisters, cupping, hot iron, etc., is based on this principle. In accordance with it I am inclined to believe that epilepsy might be cured by a mere application of a hot iron to the skin of the neck; at least I have had two guinea-pigs cured after such an application, repeated three or four times.

The operation of tracheotomy proposed by Marshall Hallhas proved successful in some cases. But it is a dangerous operation, and if it is proved that another one much slighter can produce the same good effects, it ought not to be practised.

That other operation is the cauterization of the larynx; it prevents the closure of the glottis, and thus is able to cure or to relieve epileptic patients as well as it cures some other diseases. Every learned physician knows that it is sufficient to cauterize the larynx once or twice to cure hooping cough in almost every case.

When the cause of the epileptic fits is excessive, and when the spinal cord is very excitable, to allow free breathing merely will not be sufficient to prevent the general convulsions. But their violence, if respiration is free, will be deprived of all the effect that would be produced by the excitation of black blood if breathing did not take place.

The distinction made between organic and inorganic epilepsy has not the importance that some writers seem to admit. There are alterations in the nervous system in both cases, and the only difference is that these alterations can be easily seen with the naked eye in one case and not in the other. I ought to point out that the cases of epilepsy in animals, which I have cured, although the apparent and primitive cause of the disease, *i. e.* a section of a lateral half of the spinal cord, continued to exist.

The cauterisation of the larynx on these animals was made every day, or every other day, and sometimes during two or three months. In some cases, the relief having been immediate, the cauterisation was made only twice a week. One of the animals experimented on was cured after three or four cauterisations; but the number of cauterisations necessary has been generally very much greater. When I left France in February, 1852, I had cured about a third of the animals treated by this method; and all the others, except two or three, had been very much relieved, and certainly many of them would have been cured if the treatment had been prolonged.

I know that an animal was cured, not only by the absence of spontaneous fits, but when I could not produce a fit by giving great pain. I had found that on any epileptic animal, except immediately after a paroxysm, I could very easily produce a fit by exciting pain and more particularly by pinching or burning the skin of the face or neck. So that I am authorised to believe that when a fit was not produced by pinching or burning the face, it was because epilepsy had ceased to exist.

Some physicians in this country have already tried on men the mode of treatment that I have found so successful on animals. From what I know of the results of their attempt, it seems to me that man is like animals in this respect. There has not been yet a complete curation; but, except in one case, there has been a very considerable diminution in the frequency and the intensity of the fits.

As physicians who have to treat epileptics, have not to make experiments, but to cure by making use of all the best means together, I think that the treatment of epilepsy ought not to consist merely of the cauterisation of the larynx. The plan of treatment I should suggest is the following:

1st. A cauterisation of the larynx with a strong solution of nitrate of silver, (at least 60 grains to the ounce,) every day, for at least five or sixs weeks.

2d. A cauterisation of the skin of the neck over the spine, with a hot iron, once a fortnight, for about two or three months.

3d. Exercise and gymnastics.

4th. Make use of oxide of zinc or ammoniated copper, remedies which a very respectable physician of Geneva, [Switzerland] Dr. Herpin, has found successful in many cases, when their dose has been considerable.*

5th. If in a fit of epileptic the suffocation is very considerable, the operation of tracheotomy ought then to be performed immediately.

Our author maintains that the apparantly spontaneous contractions ocurring during life and after death, are due to a stimulu sbrought in contact with the contractile tissues by means of the circulation. He has found that these contractions are much more marked during asphyxia. From this fact, and other reasons, he has been led to believe that the force of the hearts action is increased during asphyxia at least for a short time. Our author details a series of experiments tending to establish the same fact, from all of which he concludes that "black blood, by its carbonic acid, is an excitant

---

* See his admirable work: *Du Pronostic et du Traitment de l'Epilepsy.* Paris, 1852. Ouvrage couronne par l'Institut de France.

of the beatings of the heart. If, now, we adduce to these facts all those I have related in a preceding article, on the apparently spontaneous contractions in all the contractile tissues of the body, we shall have a very considerable number of facts, proving that, during asphyxia, there is an accumulation in the blood of the principle which causes these contractions. I believe that it is almost impossible to deny that this principle is the carbonic acid gas."

This book is a true exemplification of the maxim *Multum in Parvo.* J.

